# Spatio-temporal analysis of oil lake and oil-polluted surfaces from remote sensing data in one of the Libyan oil fields

**DOI:** 10.1038/s41598-020-76992-5

**Published:** 2020-11-19

**Authors:** Khalifa Abdunaser

**Affiliations:** Libyan Petroleum Institute, P. O Box 6431, Tripoli, Libya

**Keywords:** Environmental sciences, Environmental monitoring

## Abstract

The study area, which is part of the Sirt sedimentary basin in the north-central part of Libya, is characterized by natural resources of important environmental value that need special attention as they are threatened by many human activities. The focus of this study was mainly on the production of high-resolution maps of oil-contaminated surfaces, and the series time maps of events resulting from oil pollution using multi temporal satellite data and validation of the results. Digital image processing techniques were used on satellite-based sensing, whether optical or radar data, which proved to be a cost-effective way to collect information on the volume of lake water, and to assess the depth and concentration of pollution in the study area rich in lakes taken from different periods (1972 to 2006). The area of the oil-contaminated lake, called produced water, was calculated from the 1972 Landsat MSS digital satellite imagery data and was about 1.8 km^2^ and then increased to 10.7 km^2^, during 2006 from Landsat digital image TM data. The size change in this area was due to the increase of the quantities of water production that continued to increase as the oil and gas fields reached maturity. The 2019 Landsat satellite imagery reveals a drastic shrinkage in the area of the lake attributed to the suspension of the produced water pumping as well as the cycle of evaporation that resulted to the water led to a limited volume of water remaining in the lake.

## Introduction

Oil pollution in producing fields located mainly on land is often monitored by sampling of soil, water, atmosphere, and plants, to determine if they are contaminated with oil^1,2^. It is usually time consuming and more expensive, especially when applied to large or inaccessible areas. It has been found that the use of satellite images can cover large areas simultaneously and periodically in a non-destructive manner with lower costs and shorter time, and thus be a viable alternative to traditional methods based on the ground^3–5^.

Multispectral remote sensing involves the acquisition of visible, near infrared, and short-wave infrared images in several broad wavelength bands are proving invaluable in developing environmental monitoring capabilities. It has become clear no doubt that when these techniques are used, they are very well suited on a large scale in the world, especially in many projects and studies to describe and provide invaluable information on the state of the oil pits (lakes) and oil-contaminated surfaces^6–10^. In addition to^11^ who applied SIR-C/X synthetic aperture radar (SAR) data to determine oil lakes, surface roughness variation and vegetation distribution. Consequently to minimize the cost and save time, remote sensing technique in addition to GIS analysis must be first utilized before any other traditional methods.

**Figure 1:**
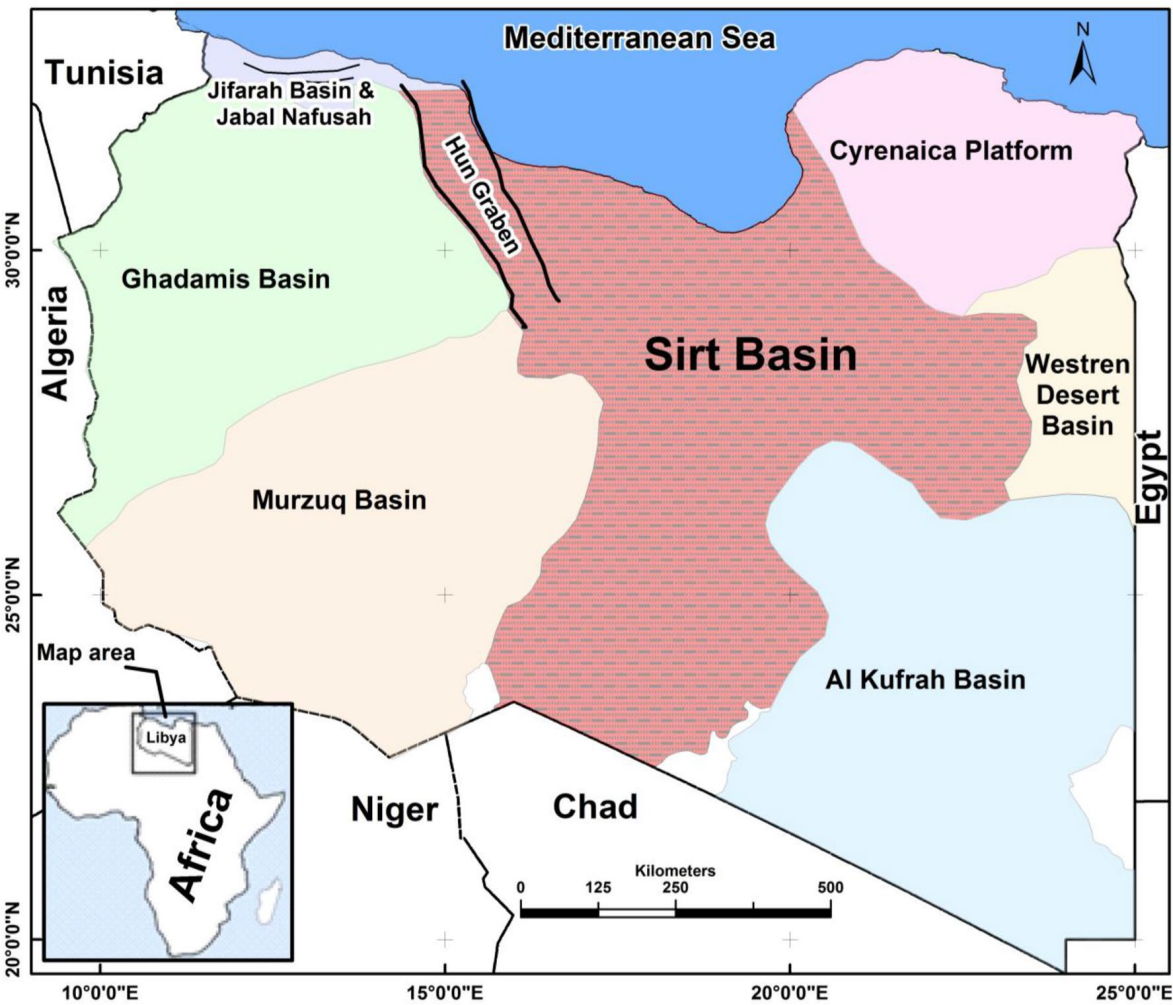
Location map of Libya (study area) showing the main sedimentary basins. *Source*: Base Cartography provided by: Libyan Government.

For the sake of confidentiality of the information, the oilfield name and exact location will not be given instead it will be mentioned as a study area located in Sirt sedimentary basin at the central north part of Libya (Fig. 1) which is an important and unique environment with valuable resources that should be carefully maintained. This area is threatened by numerous of anthropogenic activities. The most important risk is that associated with oil and gas production especially the pollution caused by produced water from oil fields.

Produced water can be defined as water present with oil or gas in ground formations and brought to the surface^12^. It is by far the largest secondary source or waste stream associated with oil and gas production. This makes managing the water produced and its environmental impacts a huge challenge for the oil industry and environmental experts.

**Figure 2:**
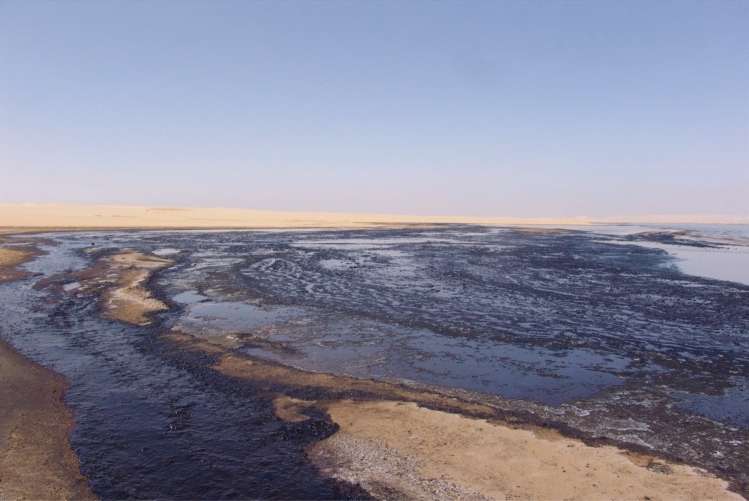
Accumulated oil at the disposal lake's bank (Photographed by the author).

The produced water (Fig. 2) is a pollutant of soil and groundwater, along with its destruction of plant life as well as the consequent erosion of topsoil. In particular, the remaining petroleum hydrocarbons can persist in the soil for decades^13,14^ and have a chronic impact on ecosystems and humans affected by soil pollution of shallow surface and shallow aquifers as well.

### Objectives

The main objective of this study is to determine the size and depth of the lake and the amount of concentration of pollutants inside the lake utilizing satellite-based sensing.

In addition, satellite data has been used for several dates of oil pollution events using indicators of change with multiple time images over the past decades.

The focus of this study was mainly on the production of high-resolution maps of oil-contaminated surfaces, and the series time maps of events resulting from oil pollution using multi temporal satellite data and validation of the results.

Through this work, the following procedures were carried out:

- Preliminary study of the satellite images and identify areas of possible oil contamination were vectorized in ArcGIS and entering attribute information in the application ArcMap.
- Perform reconnaissance of produced water body extension, depth, and locate the data on GIS to produce multi- GIS layers of the available data
- Identify latitude, longitude (in terms of x, y coordinates) of the polluting features.
- Verify the direction of flow and assess their impact on the surrounding areas.
- The satellite imagery contains all bands has been used to classify produced water bodies according to different features: i.e., extension, and depth.
- Different time series aerial and satellite imagery have been used as a base for this and used to extract features relevant to this study.
- In order to predict the future extension of the disposal lake, topographical map of the study area has been constructed. To achieve this goal the available different dataset such as the SRTM obtained elevation data on a near-global scale to generate the most complete high-resolution digital topographic database of Earth supported by local topographical maps.

## Materials and methods

The satellite images of the study area were created using a variety of digital image processing techniques to enhance three separate satellite images from different dates in the period 1972–2006 and combine them into a single composite image. The results provide a dramatic view of the lake extension.

The following main components have been covered this part of study:

1. Acquisition and processing of different remote sensing data.
2. Generation of digitally enhanced satellite images to show time series of changes to the produced water lake of the study area.
3. The used images were geographically corrected using digital image processing and geo-referenced to the same projection.
4. The images have been transferred to Arc-GIS software to produce the accurate boundary delineation of the produced water body.
5. The lake from the image were digitized and cut to perform the classification.
6. Generating of time series maps of the studied lake for different years.
7. Construction of the final maps and analyzes changes of polluted areas with the data of the previous years.

### Geology of the study area

The geological study of any area affected by oil pollution is very important in identifying the consequences of that pollution on the rock layers below or around the lake. Despite the shrinking of the produced water from these lakes, they have left their surfaces in the form of dark formations saturated with oil materials, which completely changed the characteristics of the rock in terms of porosity and cohesion, and will result in future morphological changes. Although this is outside the framework of this study which is concerned with the extension, expansion and volume of water of the lake during several years.

**Figure 3:**
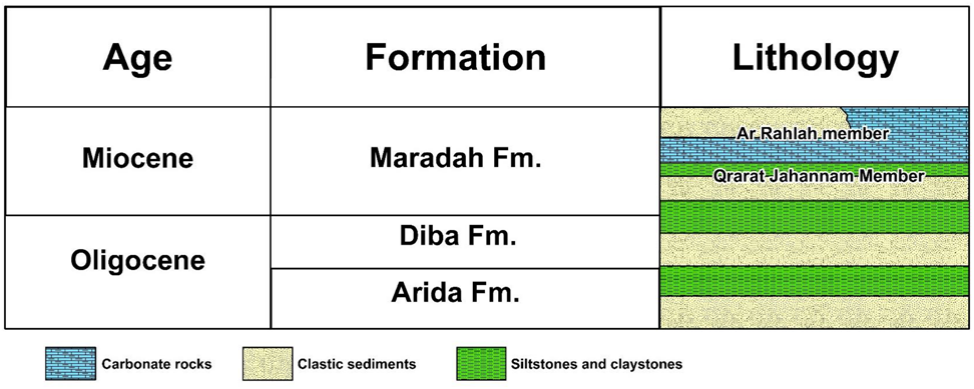
Stratigraphic section of post-Eocene sediments in the study area. Data source from published papers^16,17^.

The study area within which the produced water lake is under study is covered by the composition Maradah Formation which disconformably upon Oligocene rocks. Marine sediments in the northern part of the mapped area generally prevail over continental deposits^15^. Maradah Formation is made up of two members (Fig. 3), the lower part is Qrarat Jahannam Member, and the upper part is Ar Rahlah Member.

Ar Rahlah member consists of facies of carbonate rocks and terrigenous clastics. The facies of the carbonate rocks is characterized by a predominance of carbonate rocks over clastic rocks.

Qrarat Jahannam Member consists mostly of clastic sediments of estuarine and shallow-sea origin. In this member sands account for 30.75%, sandstones for 24.12%, calcareous sandstones for 12.21%, siltstones for 2.31%, and claystones for 22.97% by volume. The clastic component predominates over the chemogenic one.

### Data interpretation

#### Satellite imageries

This study illustrates that satellite images can be used to show what cannot be seen or measured by other traditional techniques where the changes have been measured accurately during the period from 1972 till 2006 and found to be as following.

**Figure 4:**
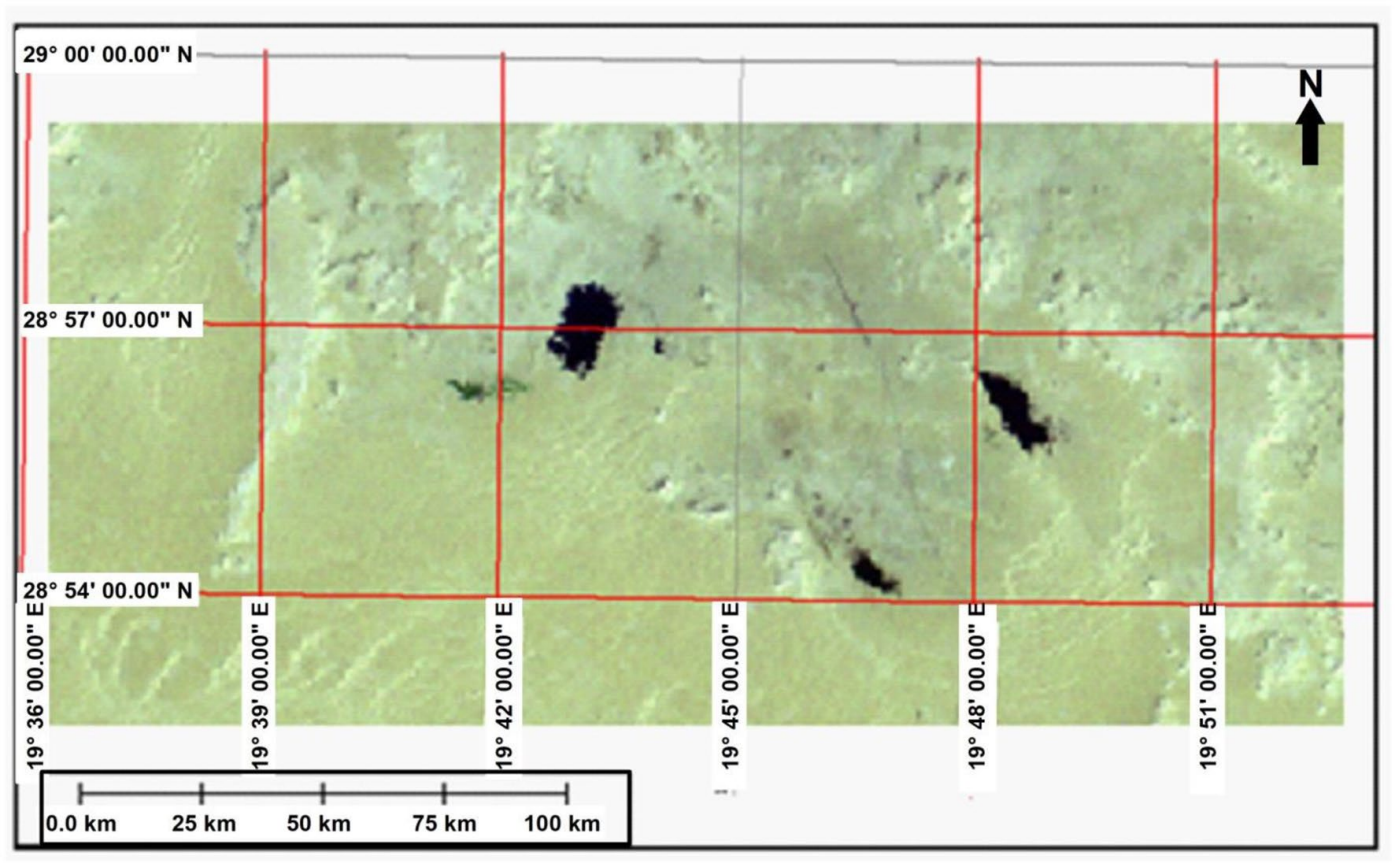
Satellite image (Landsat MSS 1972, bands 4, 3, and 2. *Source*: https://earthexplorer.usgs.gov/, public domain (Free) reproduced without permission. Generated by ArcMap software: https://desktop.arcgis.com/en).

Figure 4 is made from a sub scene of Landsat MSS acquired on 1972. MSS on the Landsat satellites each had 4 spectral bands were similar to 1, 2, 3, and 4 on Landsat 4 and 5 satellites. Two of the bands are in the visible range while 2 of them are in the reflective near-infrared. These bands have original pixel size 79 × 57 m; production systems where resampled to 60 m. The bands 4, 3, and 2 (False colour combination) were selected in this study to detect water body boundaries. The colours correspond closely to those one which could be observed from an aircraft.

The produced water appears black due to the absorption of the water by infrared wavelengths while vegetation surrounding it appears as green. Also this image shows three lakes located in the area where the lake under this study found in the north-west of the image where the dark signatures connected to some of these spots are believed to be produced water (i.e. water that comes from the reservoir together with the oil during production). The size of the lake when measured from satellite image was about 1.8 km^2^. False-colour composite imagery is therefore very sensitive to the lake water contents.

The studied lake is visible in the images as black pools. Satellite imagery helped reduce the costs of mapping these pools and quantify the level of lake expansion. The sand and gravel on the land's surface combined with oil and soot to form a layer of hardened “tar Crete” over the boundary of the area.

**Figure 5:**
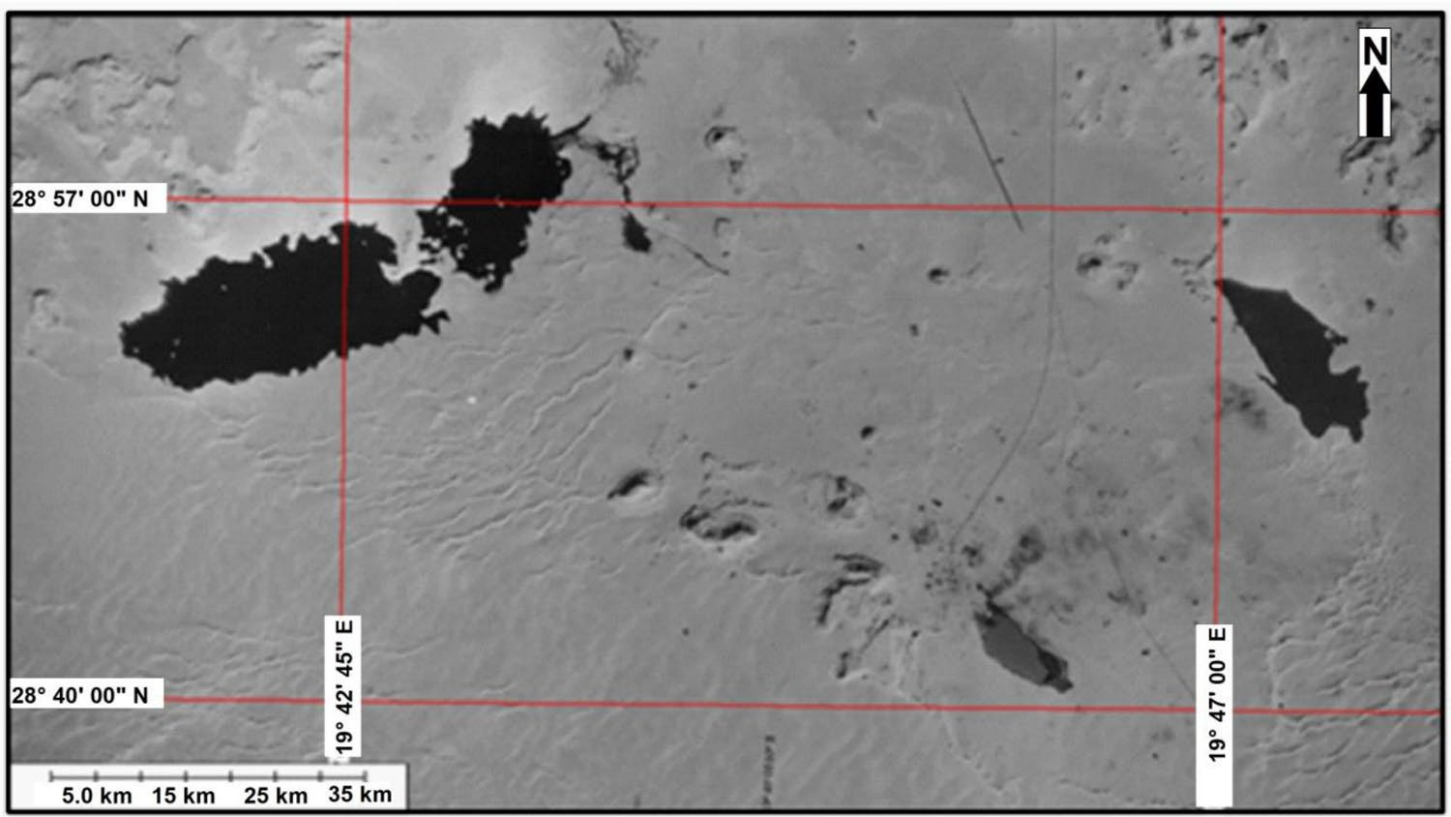
Arial Photography (1978). *Source*: Libyan Survey Dept., public domain reproduced without permission. Generated by ArcMap software https://desktop.arcgis.com/en).

Figure 5 is made from a sub scene of Aerial Photography acquired on 1978. It can be noticed from this image that the lake under study were expanded to the southwest and formed another larger lake separated by a barrier and both lakes have grown into 6 km^2^ since 1972.

**Figure 6:**
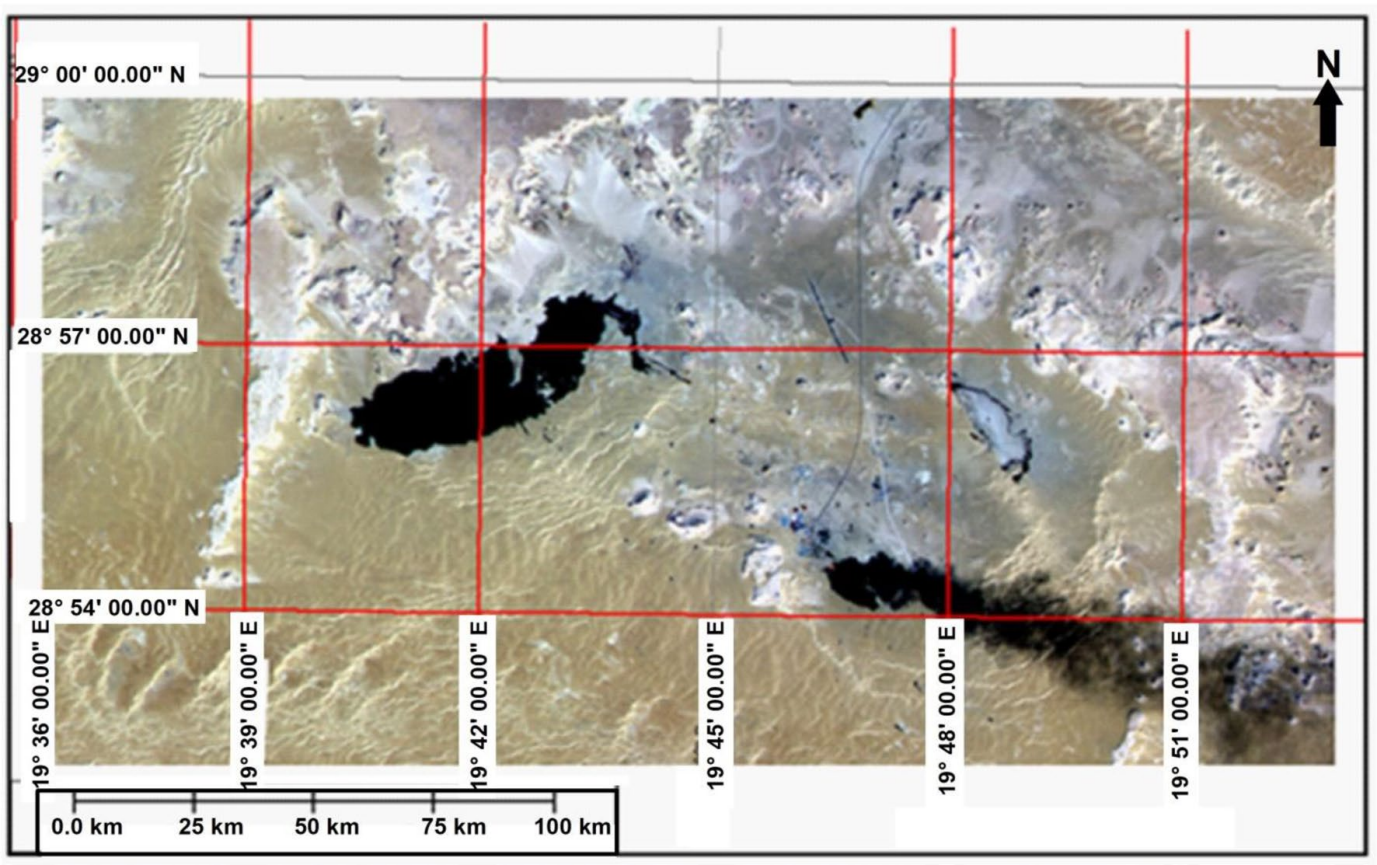
Satellite image (SPOT XS 1988). *Source*: Libyan Birouni Remote Sensing Centre., public domain reproduced without permission. Generated by ArcMap software: https://desktop.arcgis.com/en).

In 1988, the study area has been imaged by SPOT satellite image of XS mode registers light in three narrow bands (green, red, and near IR) at a nominal ground resolution of 20 m. In this image (Fig. 6) the main lake under the study became as one lake and the barrier has been reduced and submerged as a result of the water pumping. It is remained restricted only into the north central part of the lake. The bright patches in some parts of the lake are shown in this image are due to existence of some hills not submerge by produced water to. The size of the lake has been expanded to 8 km^2^.

According to this image the other lakes can be easily identified although the southern lake which located near to field camp has partially disappeared by smoke, while the one that located in the east of the field camp seems to start drying and disappearing except the Eastern boundary where still covered by water.

**Figure 7:**
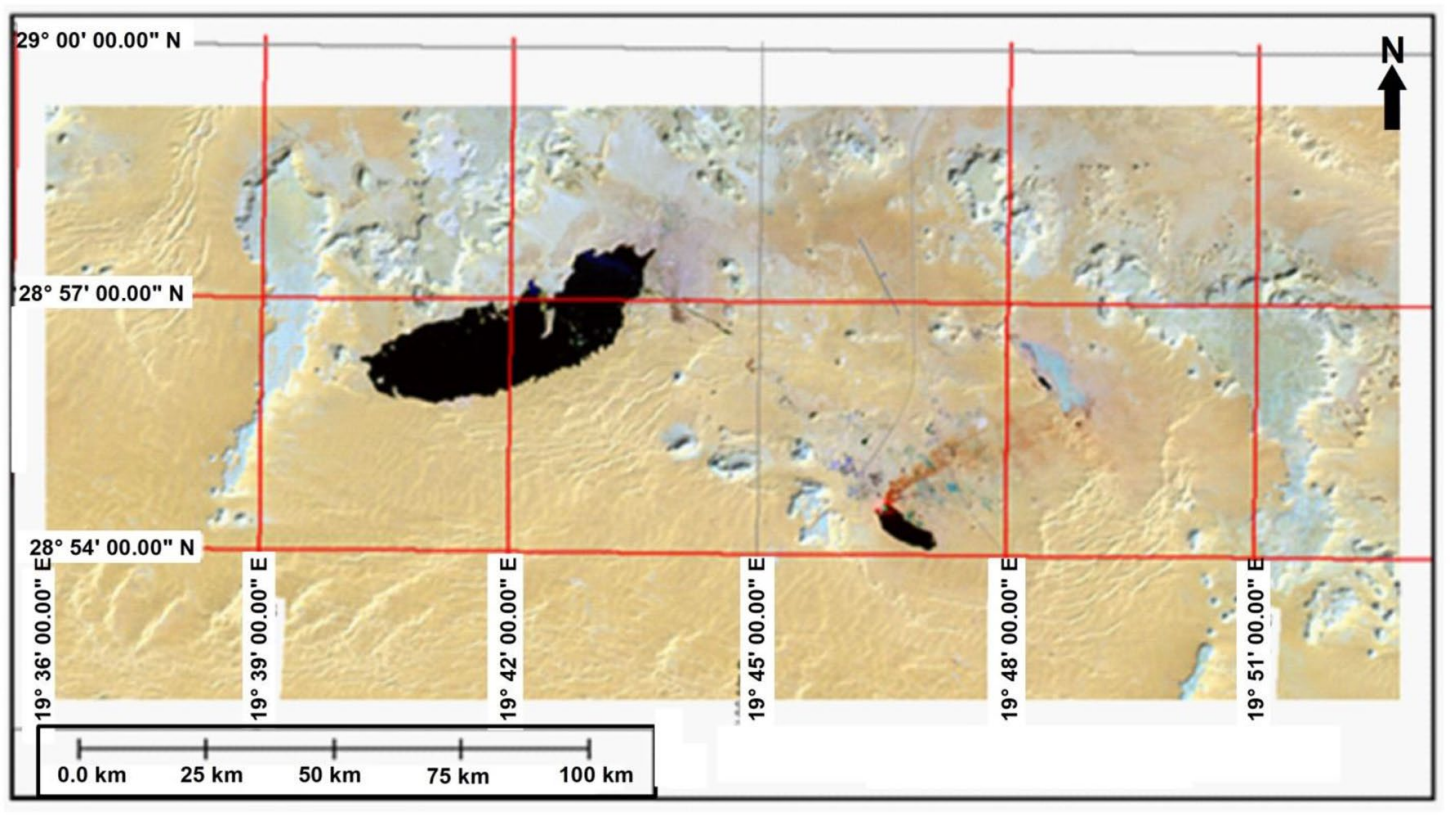
Satellite image (Landsat ETM 2004) bands 7, 4, and 2. *Source*: https://earthexplorer.usgs.gov/, public domain reproduced without permission. Generated by ArcMap software: https://desktop.arcgis.com/en).

Figure 7 shows the Landsat 7 ETM covering the study area. It was acquired in 2004. Landsat 7 Enhanced Thematic Mapper Plus (ETM+) images consist of eight spectral bands with a spatial resolution of 30 m for Bands 1 to 7 and Band 8 (panchromatic) is 15 m. This data is presented by Landsat image bands 7, 4, and 1 (False colour combination) but with the enhanced Thematic Mapper which registered big change in spatial resolution by the addition of panchromatic band of 15 m resolution. The lakes are easily recognized from this image particularly the one which appears as black spot as a result of using infrared bands that absorb the lights. Compared with the previous image acquired in 1988 by SPOT satellite the lake seems to be expanded into the west which can be indicated by the covered part of land where the top of the hill is still appearing. The barrier is more reduced and still connected to the land. The bright patches that previously mentioned are still appearing in some parts of the lake. The size of the lake has expanded to about 10 km^2^.

The other lakes can also be still easily identified where the southern lake which located near to field camp is appearing bounded in the north by a flame with arising smoke from some field wells.

While the lake is located in the east of the field camp seems to be almost dried and disappeared except a small part in the western boundary which is still covered by water.

**Figure 8:**
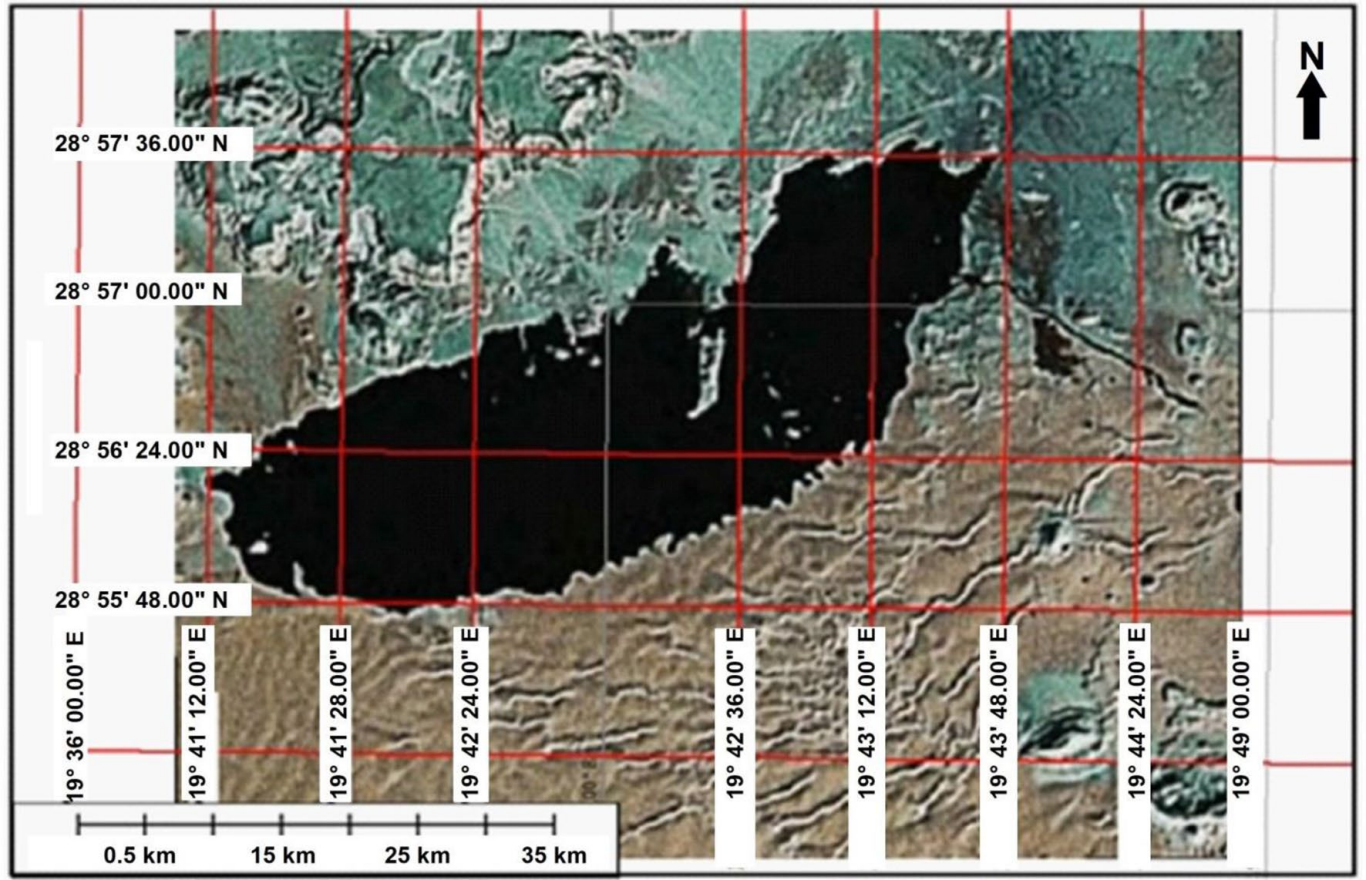
Satellite image (Landsat ETM 2006) bands 7, 4, and 1 (RGB). *Source*: https://earthexplorer.usgs.gov/, public domain reproduced without permission. Generated by ArcMap software: https://desktop.arcgis.com/en).

Figure 8 is Landsat-7 ETM image and is acquired during in 2006. It shows an enlargement of the part of the image revealing the main produced water lake under the study to show it more clearly and comparing it with its status in 2004.There is no big difference in terms of shape and size except the mentioned barrier which became an isolated island inside the lake. The lake seems to be increased in its margins and most likely expanded more to the west and the whole size of the lake increased to about 0.7 km^2^ to become about 10.7 km^2^.

**Figure 9:**
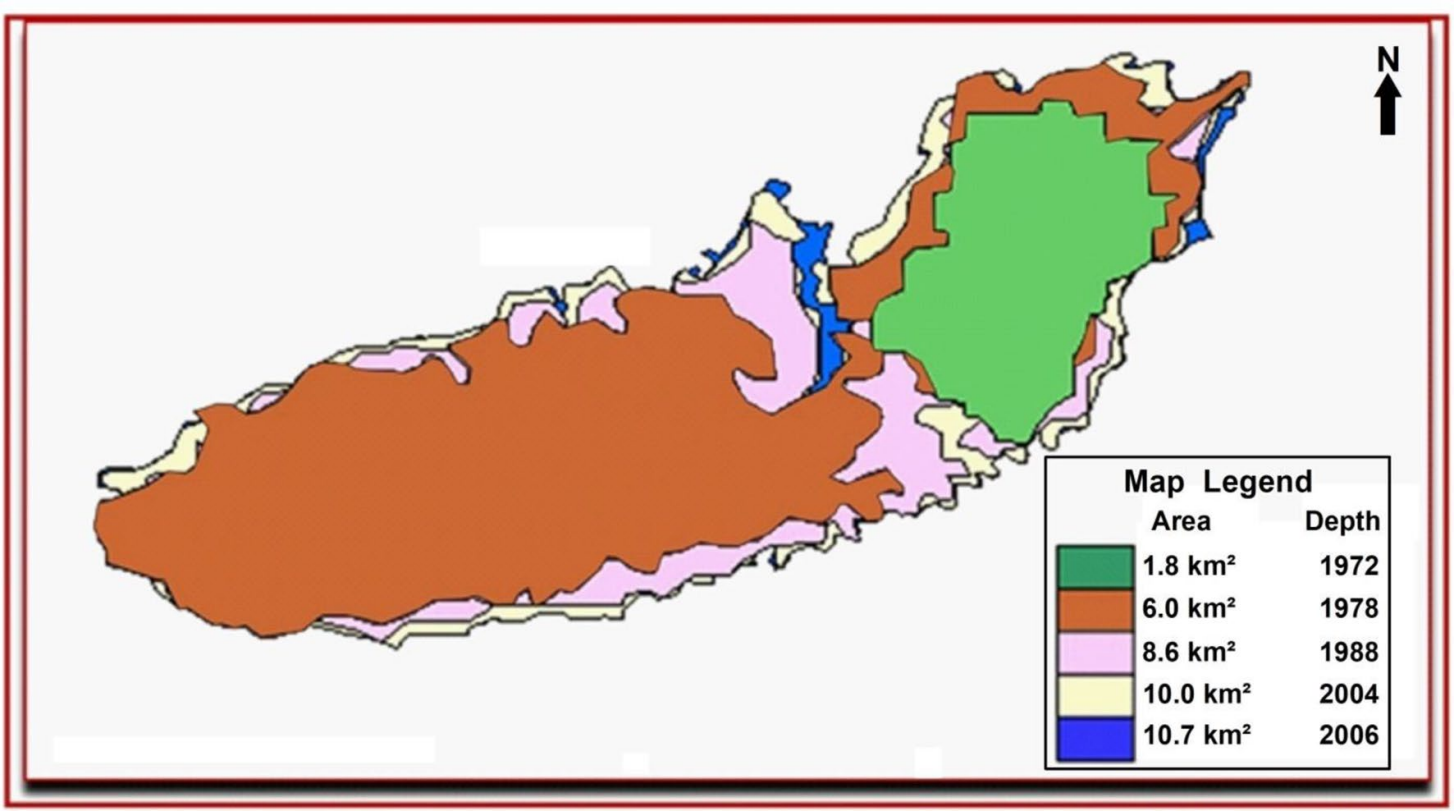
Produced water lake status (1972–2006) based on previous satellite image interpretation generated by ArcMap software: https://desktop.arcgis.com/en.

Figure 9 shows a combination map of the satellite images from 1972 to 2006 as a series time map showing the frequent change in size. From this statistic it is very evident that the lake was extended mainly from north-east to south-west and the greatest change occurred between 1972 and 1978 when the lake increased dramatically from around 1.8 km^2^ to 6 km^2^ and may be attributed to peak field production during that time.

The graph shows the effects of fluctuation in the amount of multi-year pumping (1972–2006) and subsequent return to more favorable pumping conditions.

**Figure 10:**
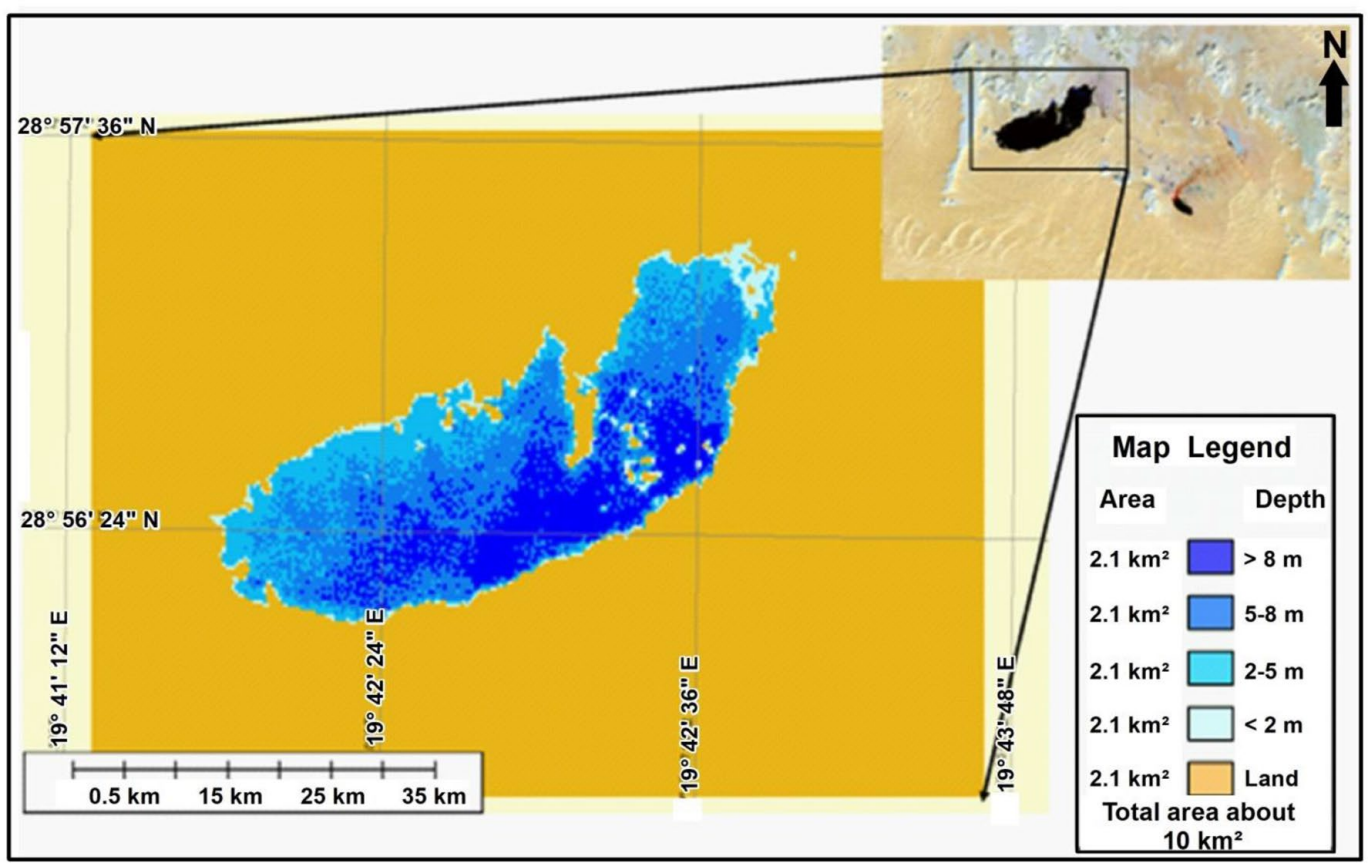
Classification map shows depths of the lake overlaid on satellite image based on previous satellite image interpretation generated by ArcMap software: https://desktop.arcgis.com/en.

Figure 10 is a classified map of the main lake under study which shows the distribution of the depths with their sizes based on a field survey measurements and extrapolated on satellite image.

This classified map reveals that the shallowest areas located at the margins of the lake with a depth of less than 2 m which increases gradually into the south central part of the lake coloured in dark blue with a depth of more than 8 m. The graph illustrates the different sizes with different depths where the largest area of the lake is about 4.2 km^2^ with a depth from 5 to 8 m while the shallowest area is about 0.6 km^2^ with a depth less than 2 m.

### Topographical relief of the site

**Figure 11:**
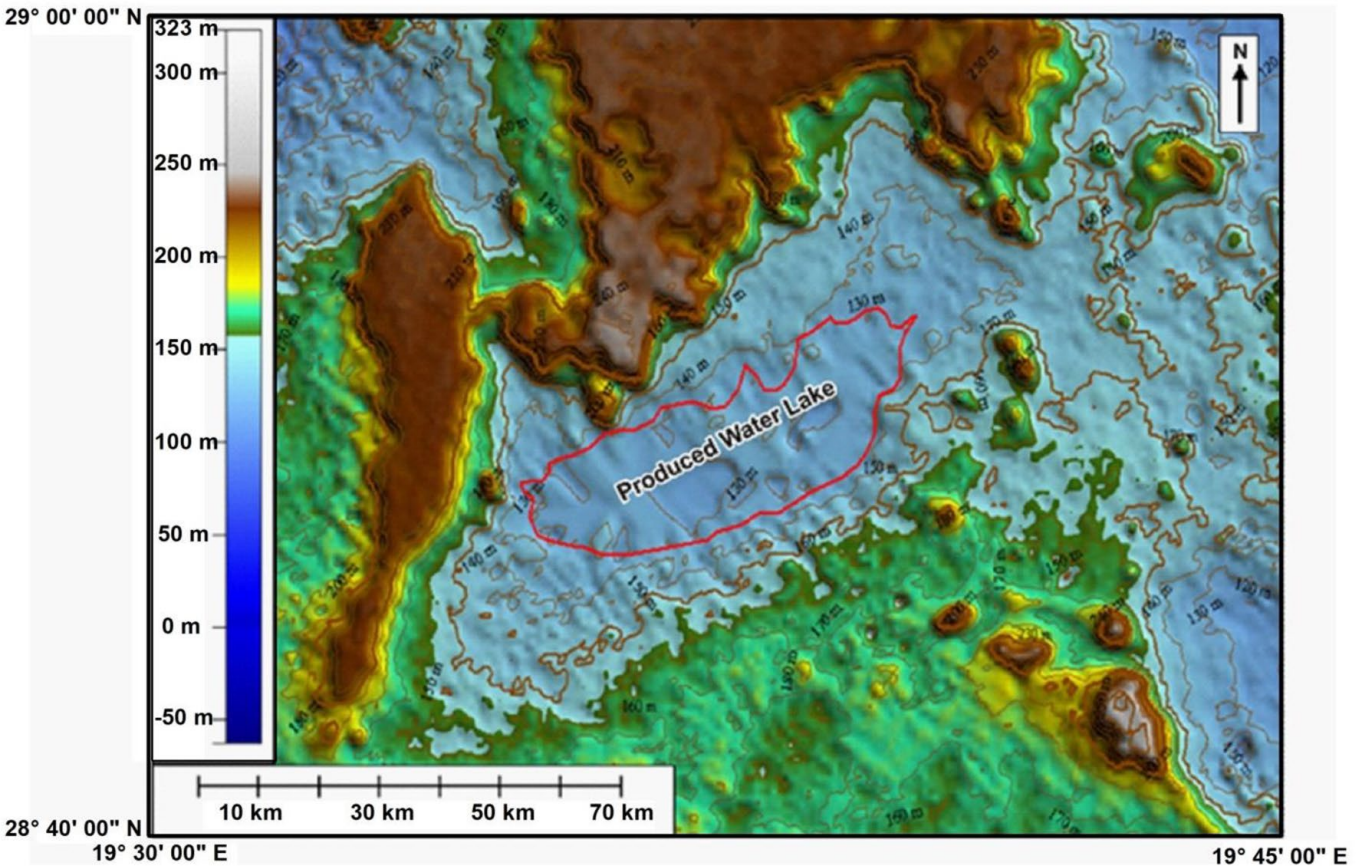
Topographical map of the study area and the surrounding areas. *Source*: GDEM (USGS-Japan Government), https://earthexplorer.usgs.gov/, public domain reproduced without permission. Generated by ArcMap software: https://desktop.arcgis.com/en.

The use of topographic map shows that the terrain of the study area ranges from 120 to 240 m above sea level (Fig. 11). The same map shows the lake and the surrounding area bordered by two relatively high areas in the north at about 240 m above sea level, which is the highest and in the south, ranging from 180 to 200 m above sea level (Fig. 11). The north-east and south-west sides of the lake are among the relatively least areas, which range between 120 and 140 m above sea level. The topographical relief of the area suggests that the proper future extension of the lake will be mainly in south western to north eastern side of the lake. In the far western side of the lake there is an elevation barrier that blocks the extension of the lake to western direction. If the lake manages to a rise more elevation, the proper future extension of the lake will be in eastern side of the lake rather than the western side. The Eastern side of the lake is lower in elevation than the western side.

## Results and discussion

This study is demonstrating lake clarity, expansion, in case of obtaining altitude information (predictable depth) can be estimated over very large areas via satellite data this level of detail is one of the benefits of this techniques.

- In 1972 the size of the lake when measured from satellite image was about 1.8 km^2^.
- 1978 the lake has risen from 1.8 to reach to 6 km^2^.
- In 1988 the study area has been imaged by SPOT satellite image and the size of the lake was expanded into 8 km^2^.
- 2004 the size of the lake was expanded into about 10 km^2^.
- In 2006, the lake was most likely expanded more to the west and the whole size increased by about 0.7 km^2^ to become about 10.7 km^2^.
  - Depths of less than two meters which represent shallow areas are located on the edge of the lake and this depth gradually increases as we head to the south-central part of the lake, which appears in dark blue with a depth of more than 8 m. The lake is characterized by difference in size or depths where areas with depths between 5 and 8 m is the largest area in the lake by about 4.2 km^2^, while the less shallow areas and not exceed 2 m deep covered an area of about 0.6 km^2^.
  - There is a possibility of expansion of the lake into the east rather than to the west.
  - This study shows that the increase in the lake occurred mainly to the north-east and south-west, the greatest between 1972 and 1978, where the size of the lake grew significantly from approximately 1.8 km^2^ to 6 km^2^, likely due to topographical factors by flooding the eastern lake and flowing horizontally into the west.
  - The volume of the lake can be measured in a certain time by multiplying the area with the average depth at that time. At 2006 the volume of the lake was as follows:
    $$ {\text{Volume 1}}\, = \,{\text{Area 1}}\, \times \,{\text{Depth 1}}\, = \,{2}.{\text{1 km}}\, \times \,{\text{8 m}}\, = \,16{,}800 {\text{m}} $$
    $$ {\text{Volume }}2\, = \,{\text{Area }}2 \times {\text{Depth }}2\, = \,4.2\,{\text{km}}^{2} \times \,6.5\,{\text{m}}\, = \,27{,}300\,{\text{m}}^{3} \,\left( {{\text{average}}\,{\text{depth}}\,{\text{of}}\,5\,{\text{to}}\,8\,{\text{m}}} \right) $$
    $$ {\text{Volume}}\,3\, = \,{\text{Area}}\,3\, \times \,{\text{Depth}}\,3\, = \,2.7\,{\text{km}}^{2} \, \times \,3.5\,{\text{m}}\, = \,9450\,{\text{million}}\,{\text{m}}^{3} \,\left( {{\text{average}}\,{\text{of}}\,3\,{\text{to}}\,5\,{\text{m}}} \right) $$
    $$ {\text{Volume}}\,4\, = \,{\text{Area}}\,4\, \times \,{\text{Depth}}\,4\, = \,0.6\,{\text{km}}^{2} \, \times \,2\,{\text{m}}\, = \,1200\,{\text{million}}\,{\text{m}}^{3} $$
    So, the total volume of the lake during 2006 is:
    $$ {\text{Total}}\,{\text{Volume}}\, = \,{\text{Volume}}\,1\, + \,{\text{Volume}}\,2\, + \,{\text{Volume}}\,3\, + \,{\text{Volume}}\,4\, = \,54,750\,{\text{m}}^{3} $$
  - The volume of the oil slick: The result of a calculation using parameters recorded during the detection (remote sensing instruments) and observation (visual) of related circumstances and conditions is only an estimation; for the existing quantity.

**Figure 12:**
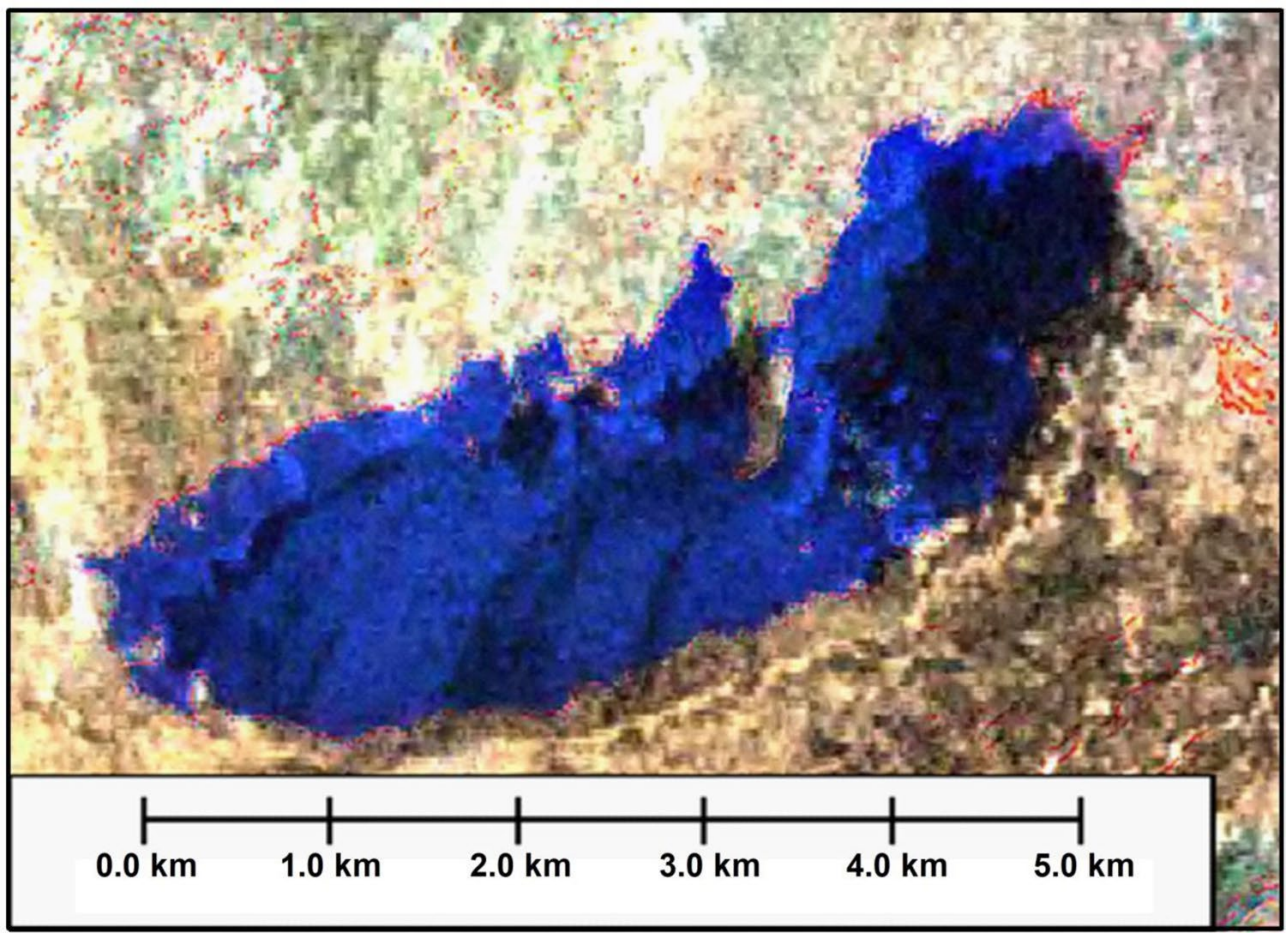
Radar image merged with Landsat7 ETM image showing the crude oil accumulated on the produced water lake and appears as black colours while water appears as blue. *Source*: https://earthexplorer.usgs.gov/, public domain reproduced without permission. Generated by ArcMap software: https://desktop.arcgis.com/en.

The oil appearance as black tends to follow a pattern as shown in Fig. 12. The thinner layers of oil, are normally at the edges of the thicker layers of oil, discontinuous true colour. It would be unusual to observe thick oil without the associated thinner oils; however, this can occur if the oil is aged and /or weathered.

We should take in account that as a result of the resolution limitation of the image which generally 15 m the small areas of less than 15 m which possibly covered with oil will appear within the overall area the image. The overall area calculations should be ‘adjusted’ to take into account the areas which characterized by clear water within the main body of the slick.

**Figure 13:**
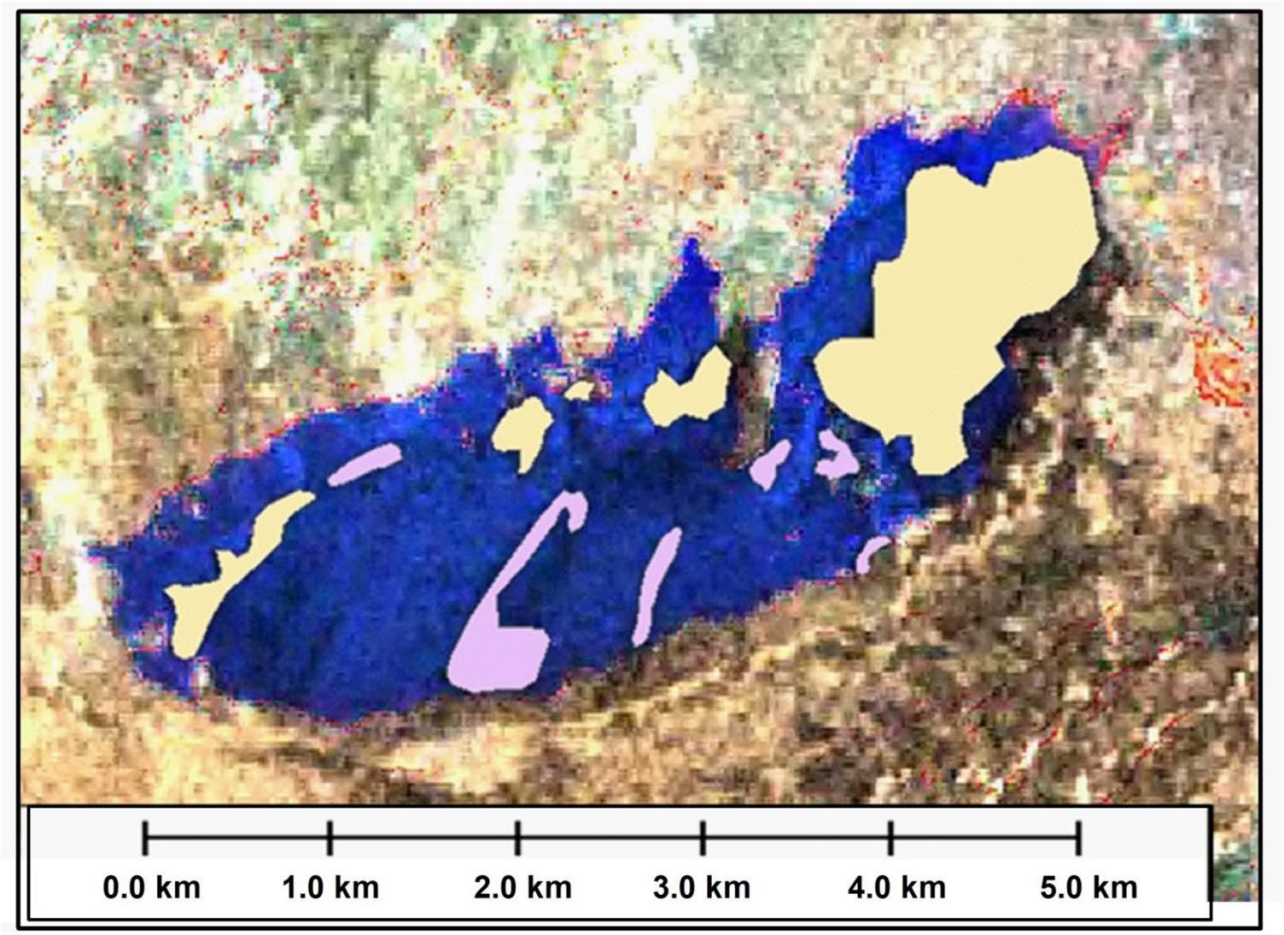
Areas were measured by drawing a polygon around the detected slick and the overall length and width have been measured and estimated visually, *Source*: https://earthexplorer.usgs.gov/, public domain reproduced without permission. Generated by ArcMap software: https://desktop.arcgis.com/en.

Usually the crude oil is characterizing by the difference in their optical density where black oils block shows all the wavelengths to the same degree but even then there are different ‘kinds of black’, while residual fuels can block all light passing through, even in thin layers. Therefore areas were measured by drawing a polygon around the detected slick and the overall length and width have been measured and estimated visually (Fig. 13) as follow:

### Most probable zone

The most probable zone consists of five crude oil slick areas (yellow colour) detected by satellite imagery equal to 2.08 km^2^ multiplied by the estimated depth of 2 cm to get an estimated volume of 2080 m^3^ multiplied by 1000 to convert to litres and divided by average of 159 to convert to barrels.

Then the probable total volume of accumulated crude oil is:

$$ (2080 \times 0.02) \times 1000/159\, = \,261.635\,{\text{barrel}}\,{\text{of}}\,{\text{oil}} $$

### Less probable zone

The less probable zone consists of six crude oil slick area (violet) equal to 0.522 km^2^, with an average depth of 0.5 cm. therefore the oil volume will be:

$$ (522\,{\text{m}}^{2} \, \times \,0.005\,{\text{m}}^{2} )\, \times \,1000/159\, = \,16.415\,{\text{barrel}}\,{\text{of}}\,{\text{oil}} $$

So that the total expected oil quantity (at least) = 261.635 + 16.415 = 278.050 barrel of oil.

However we should keep in mind that these quantities of oil often does not remain on the surface of the Lake, but moving and accumulate on the shores (margins) of the Lake. It is due to the wind forces and this has been measured by taking several samples along the shores of the lake as well as many meters away and showing the soil around the lakes (about 20 m from the edge of the lakes) is certainly affected as a result of produced water depositing.

The impact has been recognized and ensured by both, visual observations and the results of the analyses obtained for produced water and soil nearby lakes. Therefore we do not expect to get the full amounts mentioned in case we decided to retrieve it as crude oil.

Once this analysis has been concluded and the advice to the company owns the field to minimize the volume of this type of pollutants by reducing the amount of pumping produced water to this lake. A Landsat 8 OLI + TIRS (2019) of composed bands (B4, B3, B2 merged with band 8 (panchromatic) to reach a resolution of 15 m as RGB was used which showed that the size of the lake decreased drastically and left just around 3443 km^2^ compared to 10.7 km^2^ in 2006.

**Figure 14:**
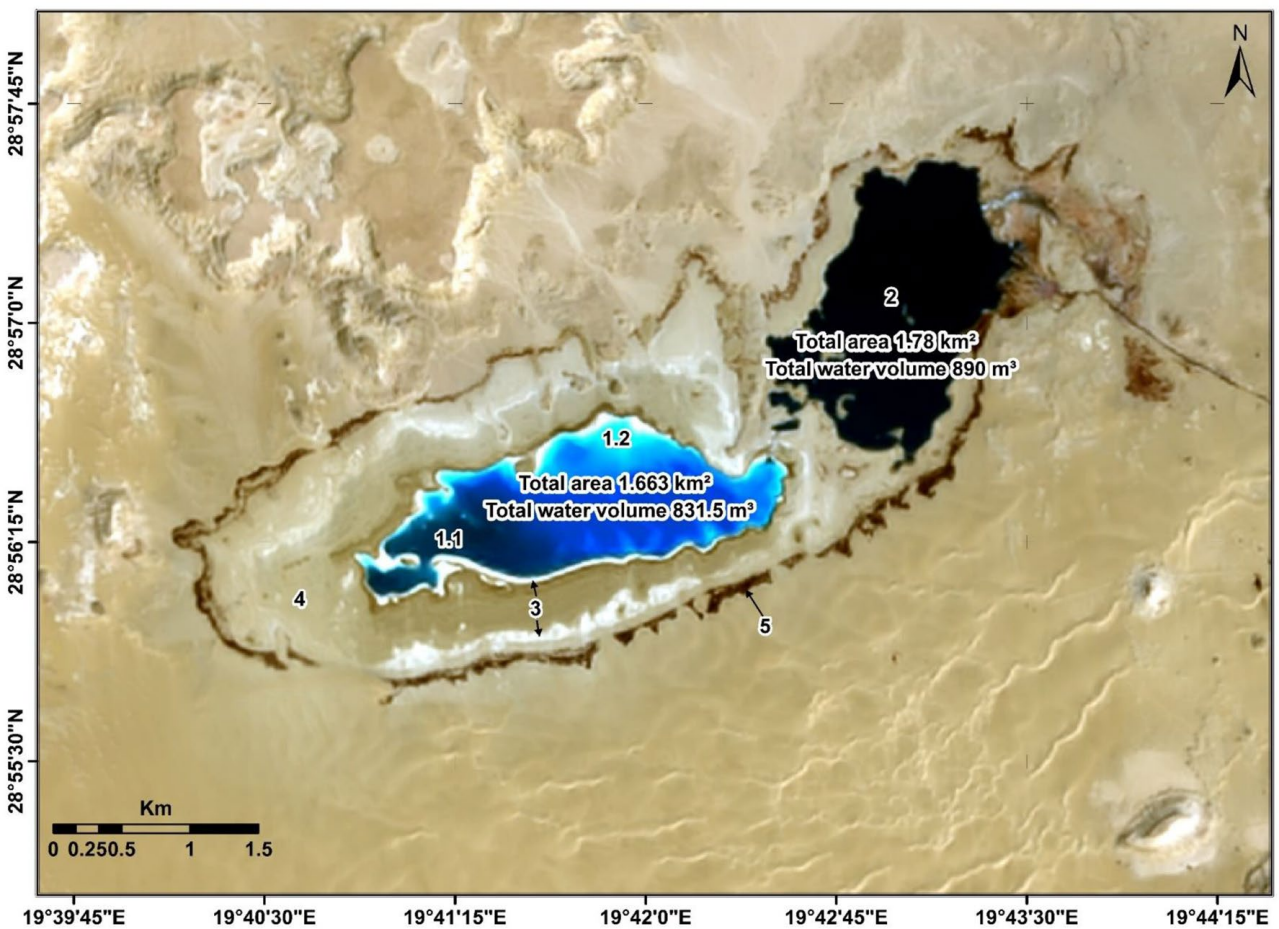
Landsat 8 OLI + TIRS (2019) of composed bands (B4, B3, B2) as RGB, *Source*: https://earthexplorer.usgs.gov/, public domain reproduced without permission. Generated by ArcMap software: https://desktop.arcgis.com/en.

As for the volume of the remaining water of the area of produced water quantity inside the lake, it does not exceed 1721.5 m^3^ that measured in this time by multiplying the area with the average depth which estimated as 0.5 m compared with 54,750 m^3^ of water that was present in the lake during year 2006. This result is because we implement the same method for lake area multiplied by the approximate average depth of 5 m at that time. This decrease is about 32% of the volume of water that covered the lake. This is evident by the interpretation of the Landsat satellite image, through which the following conclusions were reached as shown in Fig. 14:

1. The size of remaining water less polluted by oil, which appears in blue colour is only about 1.663 km^2^ whilst the water volume is about 831.5 m^3^ and the variation in the blue colour difference from very dark (1.1) to light (1.2) is due to the depth and amount of water which mixed slightly with oil.
2. As for the area of water mixed with oil, it does not exceed (2), which shows as black colour towards of the polluted lakes has become only 1.78 km^2^ and the water volume is about 890 m^3^ of the total amount of water that was present in the lake previously.
3. The areas shown in white (3) are the remainder of the amount of salts deposited in the center of the lake and on the edges.
4. It is mostly sediments of soil mixed with oil (4) has been dried and appear in the satellite image in brown to light brown inside the lake.
5. Whilst the surrounding of the lake (edges), which appears in dark brown colour (5) is the remainder of the soil, which is predominantly the concentration of oil pollutants, which occurred by pushing water currents by wind for these pollutants to settle on the edge of the lake.

From this standpoint can be used to stop the pumping for certain periods, especially in areas characterized by high temperature, such as the study area, which contributes to the drying of the lake by evaporation of water and remains only solid substances harmful to the environment and then can be removed at the final stage.

## Conclusions

From this study, it concludes that satellite images have proven successful in this form of monitoring and assessment, especially in places with broad geographical areas, and that we have been able to obtain the following results:

- By reviewing the classification map of the main components of the lake under study, the distribution of depths in their sizes was identified.
- The classified map shows that the shallowest areas are located at the margins of the lake, which is less than two meters deep and gradually rising in the south-central part, which appears in dark blue with a depth of more than 8 m. While the same map shows the different sizes of depths where the largest area of the lake, which is about 4.2 km^2^ with a depth of 5–8 m, whereas the least shallow area is about 0.6 km^2^ and a depth of less than 2 m.
- A polygon drawing method was used to measure areas affected by oil pollution and the overall length and width have been measured and estimated visually where we found that the most probable zone consists of five crude oil slick areas with the probable total volume of accumulated crude oil of 261.635 barrel of oil while the less probable zone of accumulated crude oil is 16.415 barrel of oil, therefore the total expected oil quantity is about = 261.635 + 16.415 = 278.050 barrel of oil.
- If the lake manages to a rise more elevation, the proper future extension of the lake will be in eastern side of the lake rather than the western side which it will may reach to the field facilities. This because the eastern side of the lake is lower in elevation than the western side.
- It is clear from this study that if we stop the pumping for certain periods, especially in areas characterized by high temperature, such as the study area, which contributes to the drying of the lake by evaporation of water and remains only solid substances harmful to the environment and then can be removed at the final stage.This is evident by the interpretation of the Landsat satellite image, which showed this decrease is about 32% of the lake area compared that was present during year 2006.

## References

[1] Ren L, Huang TL (2000). Contamination of soils by petroleum. Agro-environ. Prot..

[2] Zhu L, Zhao X, Lai L, Wang J, Jiang LE (2013). Soil TPH concentration estimation using vegetation indices in an oil polluted area of Eastern China. PLoS ONE.

[3] Austin JM, Mackey BG, Van Niel KP (2003). Estimating forest biomass using satellite radar: an exploratory study in a temperate Australian *Eucalyptus* forest. For. Ecol. Manag..

[4] Kooistra L, Salas EAL, Clevers JGPW, Wehrens R, Leuven R (2004). Exploring field vegetation reflectance as an indicator of soil contamination in river floodplains. Environ. Pollut..

[5] Mason DC, Davenport IJ, Neal JC, Schumann GJP, Bates PD (2012). Near real-time flood detection in urban and rural areas using high-resolution synthetic aperture radar images. IEEE Trans. Geosci. Remote Sens..

[6] Ajmi, D., Misak, F., Khalaf, F. I., Al-Sudairawi, M. & Al-Dousari, A. M. Damage Assessment of the Desert and Coastal Environment of Kuwait by Remote Sensing. *Kuwait Institute for Scientific Research, Report KISR 4405, Kuwait* (1994).

[7] El-Baz, F., Abuelgasim, A., Koch, M., Pax-Lenney, M., Lambin, E., Al-Doasari, A., Marr, P., Ryherd, S. & Morency, R. Detection by satellite images of environmental change due to the Gulf War. In The Gulf War and the Environment (eds. El-Baz, F., Makharita, R. M.) 1–24 (Gordon and Breach SciPubl, Lausanne 1994).

[8] Kwarteng AY, Al-Ajmi D (1997). Satellite Remote Sensing Applications in the State of Kuwait.

[9] Koch M, El-Baz F (1998). Identifying the effects of the Gulf War on the geomorphic features of Kuwait by remote sensing and GIS. PE&RS.

[10] Kwarteng AY, Chavez PS (1998). Change detection study of Kuwait City and environs using multi-temporal Landsat Thematic Mapper data. IJRS.

[11] Dobson, M. C., Kwarteng, A. Y. & Ulaby, F. T. Use of SIR-C/XSAR to monitor environmental damages of the 1991 Gulf War in Kuwait. In *Proc of the 1st Saudi-Japanese symposium on remote sensing application, Riyadh, Saudi Arabia, 19 to 21 October, * 105-I (1997).

[12] Clark CE, Veil JA (2009). Produced Water Volumes and Management Practices in the United States[external site], ANL/EVS/R-09/1, Prepared by the Environmental Science Division.

[13] Culbertson JB, Valiela I, Pickart M, Peacock EE, Reddy CM (2008). Long-term consequences of residual petroleum on salt marsh grass. J. Appl. Ecol..

[14] Peterson CH, Rice SD, Short JW, Esler D, Bodkin JL (2003). Long-term ecosystem response to the Exxon Valdez oil spill. Science.

[15] Domácí L (1985). Sheet Bi’r Zaltan (NH 34–14), Geological Map of Libya, Scale 1:250,000.

[16] Barr F.T. & Weegar, A.A. Stratigraphic nomenclature of the Sirte Basin, Libya. *The Petroleum Exploration Society of Libya*, p. 179 (1972).

[17] Hallett D (2002). Petroleum Geology of Libya.

